# Use of gold nanoparticles on graphite electrodes functionalized with poly (4-aminophenol) in the development of a bioelectrode for hepatitis B

**DOI:** 10.1186/1753-6561-8-S4-P57

**Published:** 2014-10-01

**Authors:** Larissa Caetano, Kellen Costa, Thalles Silva, Lídia Dias, Vinícius Rodovalho, João Marcos Madurro, Ana Graci Brito-Madurro

**Affiliations:** 1 Instituto de Genética e Bioquímica da Universidade Federal de Uberlândia, Uberlândia, Minas Gerais, 38400-426, Brazil; 2 Instituto de Química da Universidade Federal de Uberlândia, Uberlândia, Minas Gerais, 38400-426, Brazil

## Background

Hepatitis B is an infectious disease of the liver, highly prevalent in society, caused by hepadnavirus (HBV), which affects approximately 350 million individuals worldwide [1]. It is estimated that the number of deaths from the disease is in the range of 2000 to 4000 per year, mainly by hepatic cirrhosis and hepatocellular carcinoma [2]. Due to the fact that in adults in most cases the initial infection shows no symptoms, the diagnosis is difficult and late.

The development of biosensors for disease diagnosis has been a much valued currently, and in this area there are already several innovative research. Among the various detection techniques used in these sensors, due to the low cost and high sensitivity, electrochemical detection has been widely used [3]. Some polymers have high applicability in this area, because through chemical affinity can immobilize the molecule that will bind to its target. Studies realized by our group [4] indicates that the poly(4-aminophenol) electrodeposited in acid medium is interesting for the immobilization of oligonucleotides. Polymer embedded with metal nanoparticles provided the suitable microenvironment for biomolecules, since improved the electron transfer with the electrode surfaces, resultant in enhanced sensing performance [5].

In this work, specific graphite electrode modified with poly(4-aminophenpol)/gold nanoparticles was sensibilized with specific oligonucleotide for the detection of hepatitis B virus and compared to the bioelectrode in the absence of the gold nanoparticles.

## Methods

The monomer solution was prepared in 0.5 mol.l^-1 ^HClO_4 _and the electropolymerization was carried out by cyclic voltammetry in a three compartment cell using a CH Instruments potentiostat model 420A. Gold nanoparticles (1:3 solute/solvent) was added onto graphite electrode containing poly(4-aminophenol). Then, the solution containing the HBV oligonucleotide (0,414 mg.mL^-1^) was added onto poly(4-aminophenol) with gold nanoparticles. Other experiments were conducted in the absence of the gold nanoparticles. The detection of the immobilization of oligonucleotides was carried out by differential pulse voltammetry in a electrochemical cell of one compartment. The oxidation peak of guanosine present in the oligonucleotide was monitored in the potential of +0.9 V (vs. Ag/AgCl).

## Results and conclusions

It was possible the immobilization and detection of the specific probe for the hepatitis B virus onto graphite electrode modified with poly(4-aminophenpol)/gold nanoparticles using linear voltammetry. It was observed a peak in +0.94V attributed to guanosine monophosphate, an usual biomarker for biosensor. The amplitude of the current signal increased about 100% when compared to the modified electrode in the absence of gold nanoparticle. From these data, it was concluded that the use of these nanoparticles was able to increase the reactivity between the polymer and the specific oligonucleotide, which demonstrates a potential use of these platform for the development of biosensors.
